# Supplementary material for: High Contiguity de novo Genome Sequence Assembly of Trifoliate Yam (Dioscorea dumetorum) Using Long Read Sequencing
Source: Genes (Basel). 2020 Mar 4;11(3):274. doi: 10.3390/genes11030274 (PMC7140821; doi:10.3390/genes11030274)
Supplement: Supplementary file 1 [file genes-11-00274-s001.zip › x_supplement-files_yamGS-corrected-proof/File S12_corrected-updated.pdf]

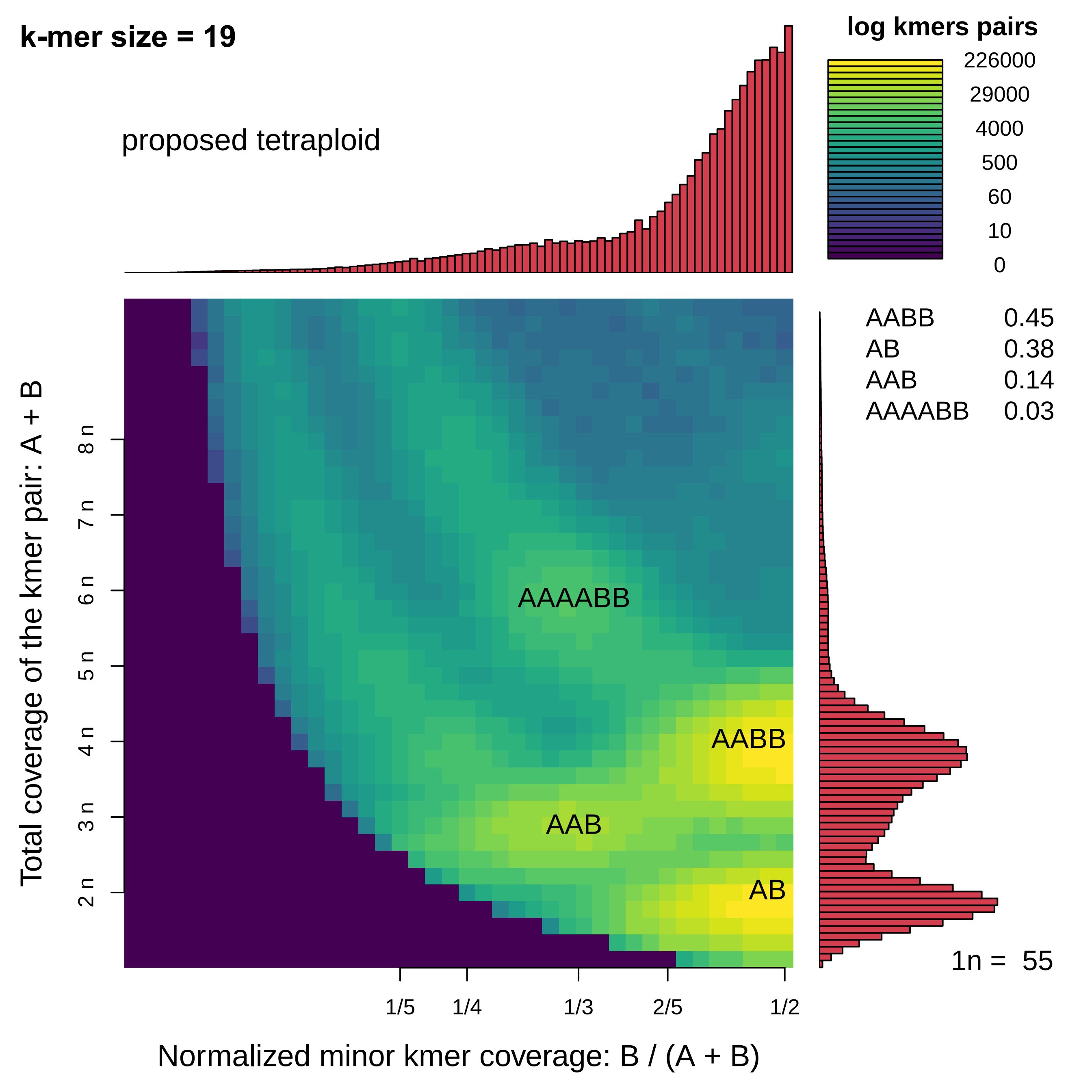
k-mer size = 19
proposed tetraploid
log kmers pairs
226000
29000
4000
500
60
10
0
AABB 0.45
AB 0.38
AAB 0.14
AAAABB 0.03
AAAABB
AABB
AAB
AB
1n = 55
Total coverage of the kmer pair: A + B
2 n
3 n
4 n
5 n
6 n
7 n
8 n
1/5
1/4
1/3
2/5
1/2
Normalized minor kmer coverage: B / (A + B)

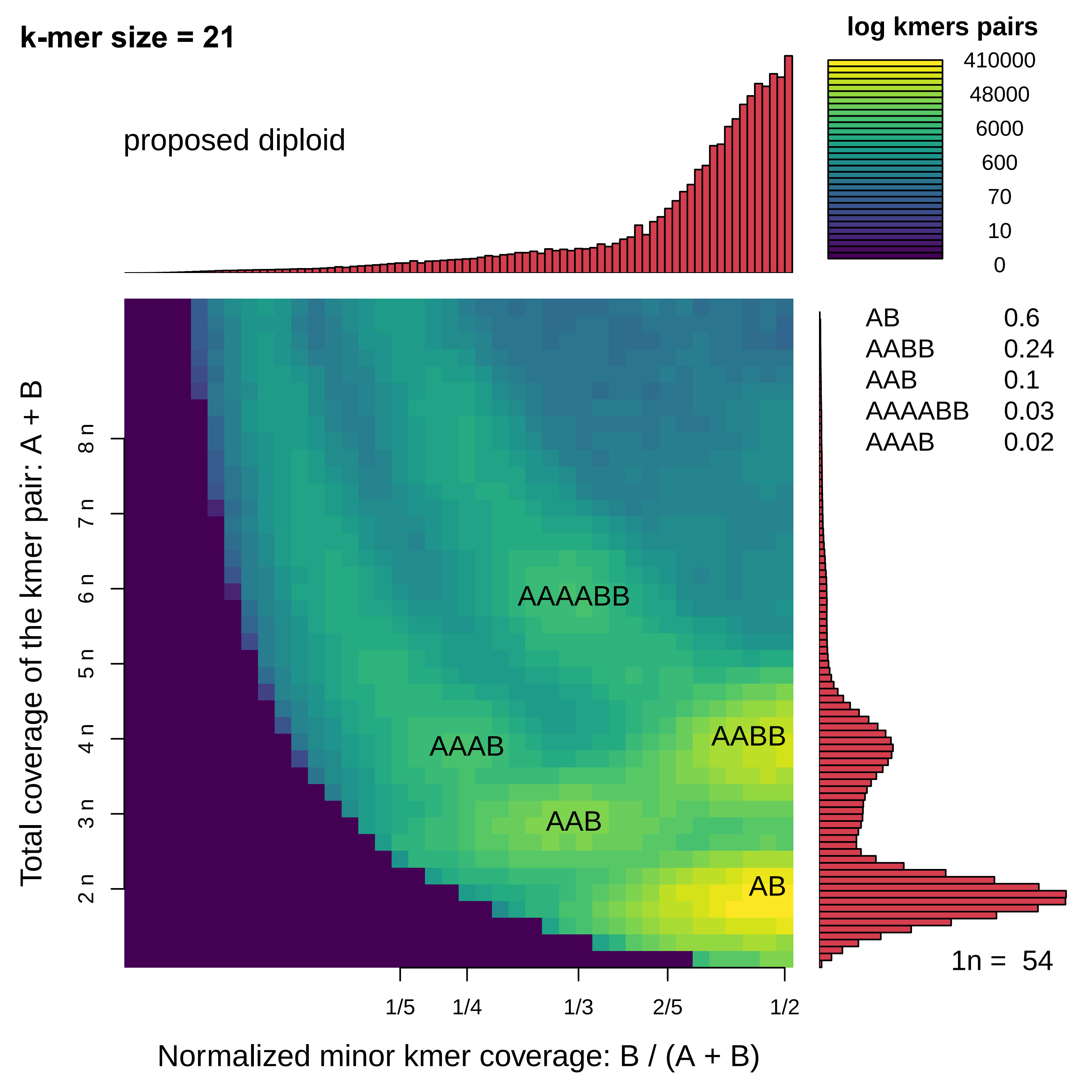
k-mer size = 21
log kmers pairs
410000
48000
6000
600
70
10
0
proposed diploid
AB 0.6
AABB 0.24
AAB 0.1
AAAABB 0.03
AAAB 0.02
AAAABB
AAAB
AABB
AAB
AB
1n = 54
Total coverage of the kmer pair: A + B
8 n
7 n
6 n
5 n
4 n
3 n
2 n
1/5
1/4
1/3
2/5
1/2
Normalized minor kmer coverage: B / (A + B)

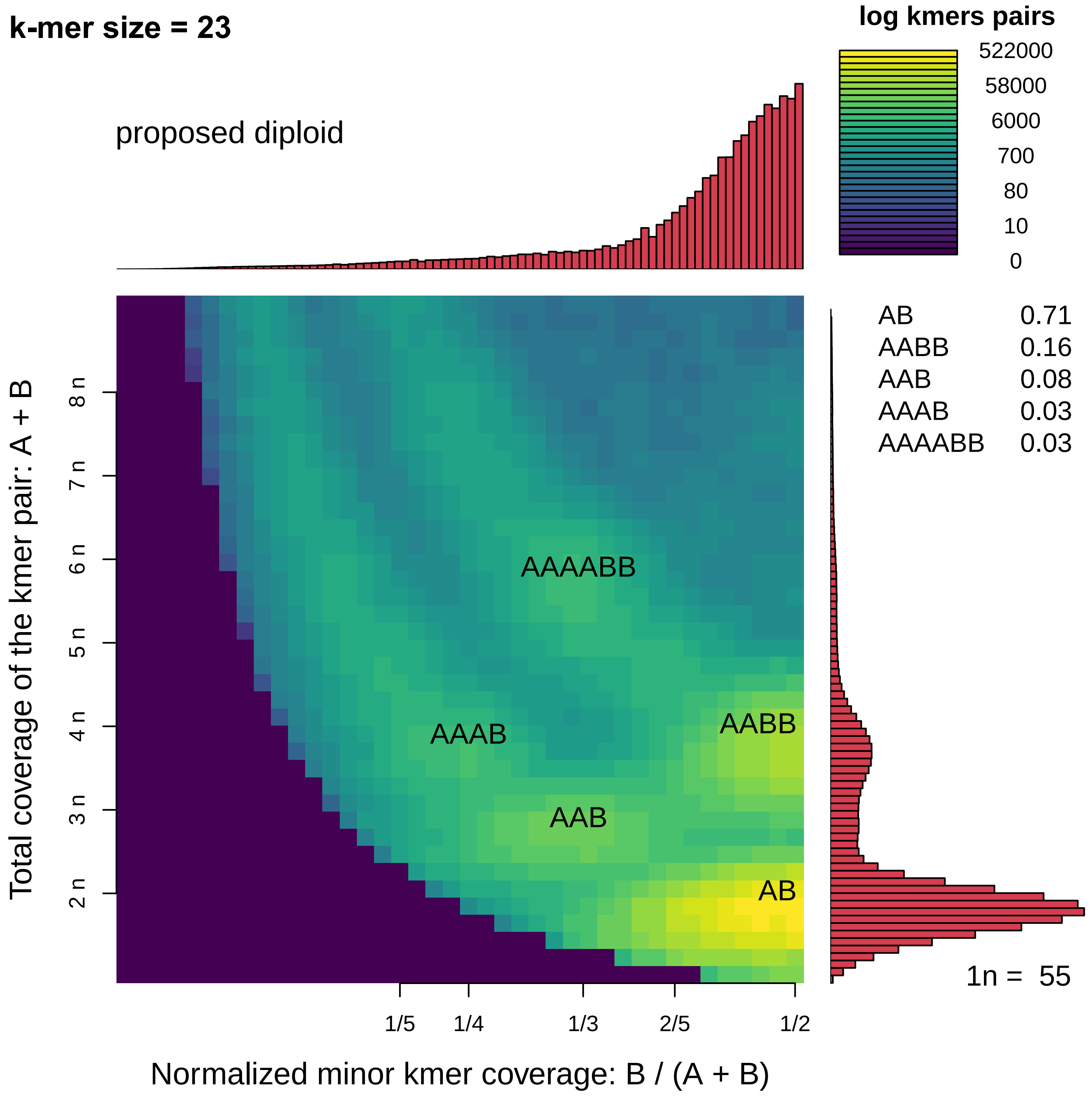
k-mer size = 23
proposed diploid
log kmers pairs
522000
58000
6000
700
80
10
0
AB 0.71
AABB 0.16
AAB 0.08
AAAB 0.03
AAAABB 0.03
AAAABB
AAAB
AABB
AAB
AB
1n = 55
Total coverage of the kmer pair: A + B
2 n
3 n
4 n
5 n
6 n
7 n
8 n
1/5
1/4
1/3
2/5
1/2
Normalized minor kmer coverage: B / (A + B)

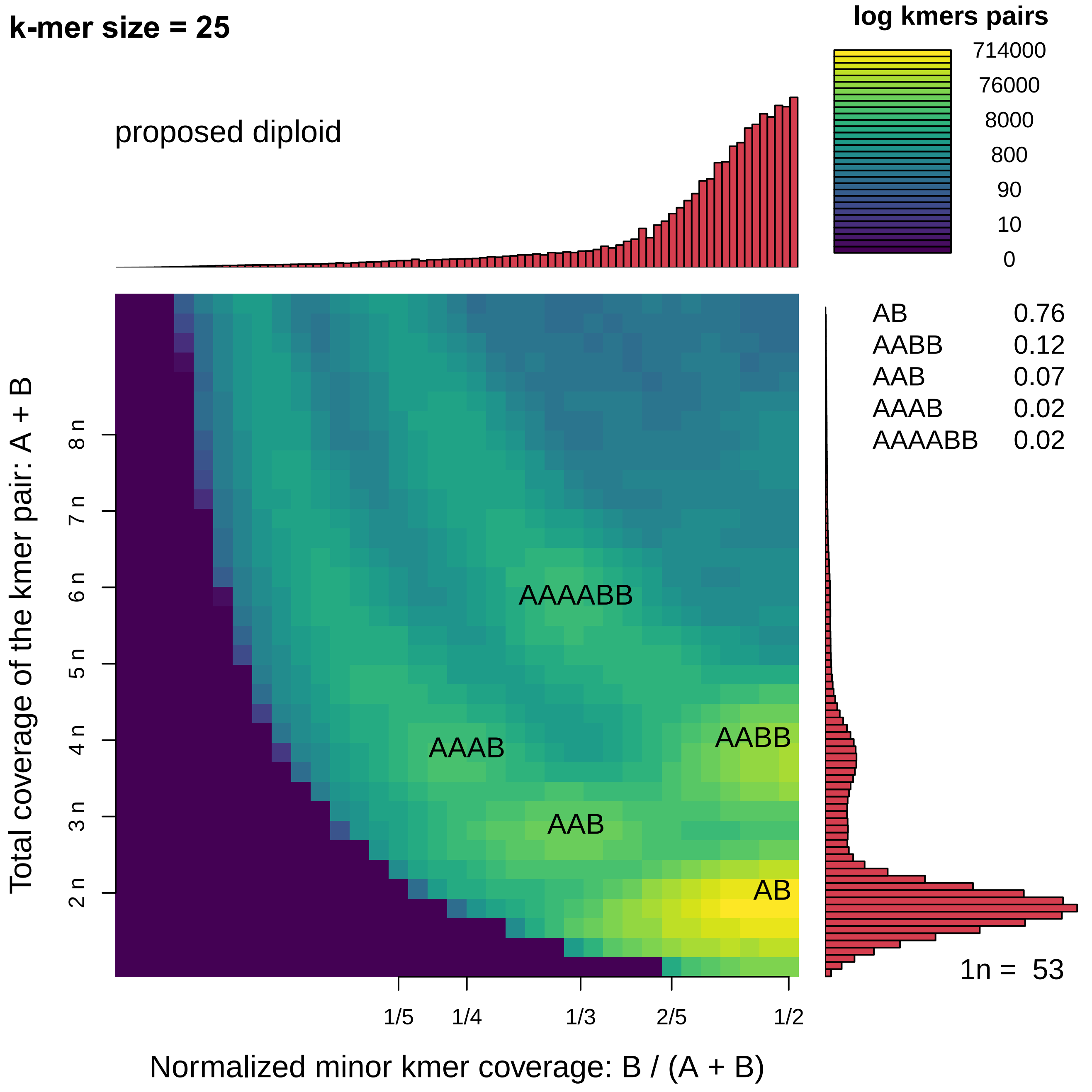

k-mer size = 25
log kmers pairs
714000
76000
8000
800
90
10
0
proposed diploid
AB 0.76
AABB 0.12
AAB 0.07
AAAB 0.02
AAAABB 0.02
AAAABB
AAAB
AABB
AAB
AB
1n = 53
Total coverage of the kmer pair: A + B
2 n
3 n
4 n
5 n
6 n
7 n
8 n
1/5
1/4
1/3
2/5
1/2
Normalized minor kmer coverage: B / (A + B)
