# Supplementary figures and images for: High Contiguity de novo Genome Sequence Assembly of Trifoliate Yam (Dioscorea dumetorum) Using Long Read Sequencing
Source: Genes (Basel). 2020 Mar 4;11(3):274. doi: 10.3390/genes11030274 (PMC7140821; doi:10.3390/genes11030274)

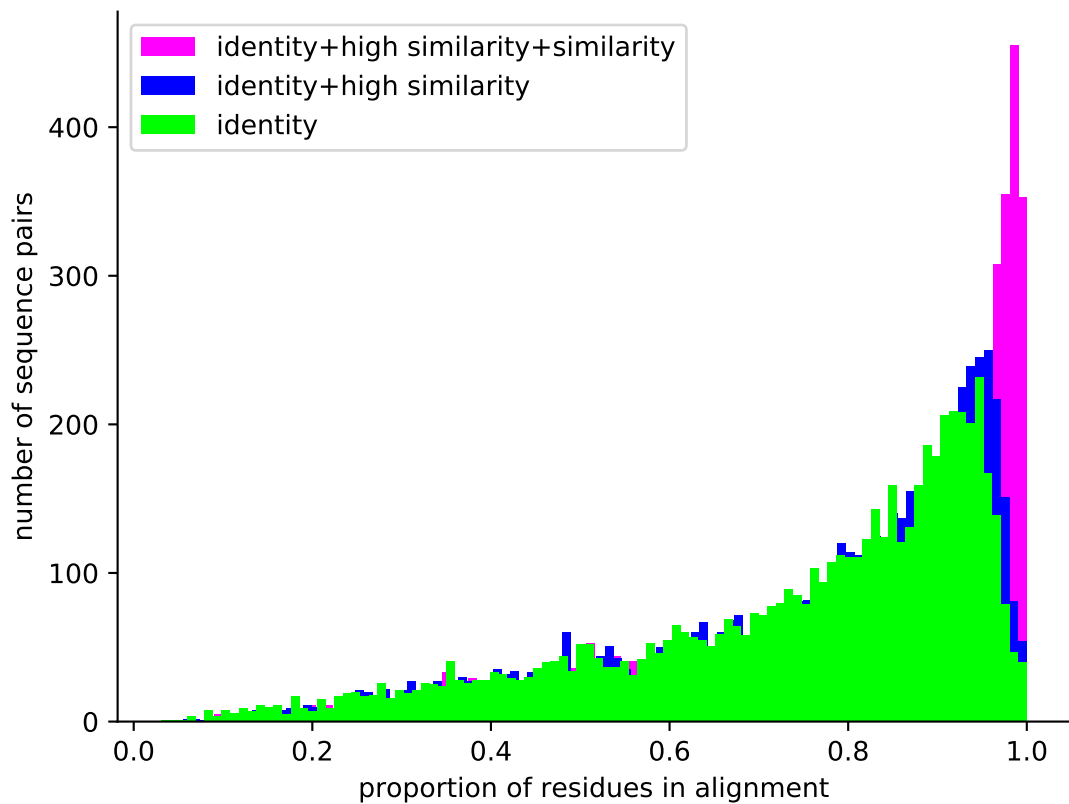

Supplement: Supplementary file 1 [file genes-11-00274-s001.zip › x_supplement-files_yamGS-corrected-proof/File S8.pdf]

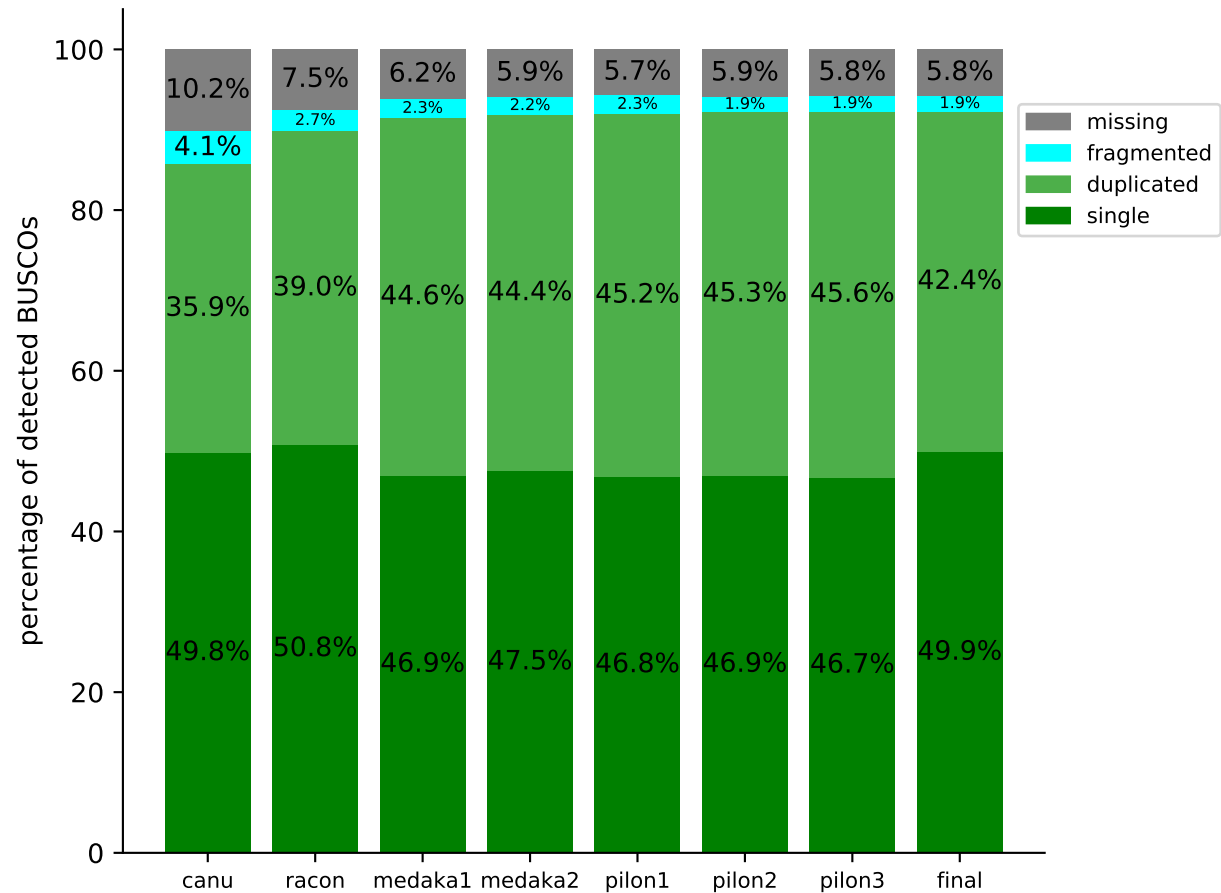

Supplement: Supplementary file 1 [file genes-11-00274-s001.zip › x_supplement-files_yamGS-corrected-proof/File S2.pdf]
